# Green tea polyphenol treatment is chondroprotective, anti-inflammatory and palliative in a mouse posttraumatic osteoarthritis model

**DOI:** 10.1186/s13075-014-0508-y

**Published:** 2014-12-17

**Authors:** Daniel J Leong, Marwa Choudhury, Regina Hanstein, David M Hirsh, Sun Jin Kim, Robert J Majeska, Mitchell B Schaffler, John A Hardin, David C Spray, Mary B Goldring, Neil J Cobelli, Hui B Sun

**Affiliations:** Department of Orthopaedic Surgery, Albert Einstein College of Medicine, 1300 Morris Park Ave, Bronx, NY 10461 USA; Department of Radiation Oncology, Albert Einstein College of Medicine, 1300 Morris Park Ave, Bronx, NY 10461 USA; Department of Neuroscience, Albert Einstein College of Medicine, 1410 Pelham Pkwy S., Bronx, NY 10461 USA; Department of Biomedical Engineering, The City College of New York, 160 Convent Ave, New York, NY 10031 USA; Tissue Engineering, Regeneration and Repair Program, Hospital for Special Surgery, 535 East 70th Street, New York, NY 10021 USA

## Abstract

**Introduction:**

Epigallocatechin 3-gallate (EGCG), a polyphenol present in green tea, was shown to exert chondroprotective effects *in vitro*. In this study, we used a posttraumatic osteoarthritis (OA) mouse model to test whether EGCG could slow the progression of OA and relieve OA-associated pain.

**Methods:**

C57BL/6 mice were subjected to surgical destabilization of the medial meniscus (DMM) or sham surgery. EGCG (25 mg/kg) or vehicle control was administered daily for 4 or 8 weeks by intraperitoneal injection starting on the day of surgery. OA severity was evaluated using Safranin O staining and Osteoarthritis Research Society International (OARSI) scores, as well as by immunohistochemical analysis to detect cleaved aggrecan and type II collagen and expression of proteolytic enzymes matrix metalloproteinase 13 (MMP-13) and A disintegrin and metalloproteinase with thrombospondin motifs 5 (ADAMTS5). Real-time PCR was performed to characterize the expression of genes critical for articular cartilage homeostasis. During the course of the experiments, tactile sensitivity testing (von Frey test) and open-field assays were used to evaluate pain behaviors associated with OA, and expression of pain expression markers and inflammatory cytokines in the dorsal root ganglion (DRG) was determined by real-time PCR.

**Results:**

Four and eight weeks after DMM surgery, the cartilage in EGCG-treated mice exhibited less Safranin O loss and cartilage erosion, as well as lower OARSI scores compared to vehicle-treated controls, which was associated with reduced staining for aggrecan and type II collagen cleavage epitopes, and reduced staining for MMP-13 and ADAMTS5 in the articular cartilage. Articular cartilage in the EGCG-treated mice also exhibited reduced levels of *Mmp1, Mmp3, Mmp8, Mmp13,Adamts5*, interleukin 1 beta (*Il1b*) and tumor necrosis factor alpha (*Tnfa*) mRNA and elevated gene expression of the MMP regulator Cbp/p300 interacting transactivator 2 (*Cited2*). Compared to vehicle controls, mice treated with EGCG exhibited reduced OA-associated pain, as indicated by higher locomotor behavior (that is, distance traveled). Moreover, expression of the chemokine receptor *Ccr2* and proinflammatory cytokines *Il1b* and *Tnfa* in the DRG were significantly reduced to levels similar to those of sham-operated animals.

**Conclusions:**

This study provides the first evidence in an OA animal model that EGCG significantly slows OA disease progression and exerts a palliative effect.

**Electronic supplementary material:**

The online version of this article (doi:10.1186/s13075-014-0508-y) contains supplementary material, which is available to authorized users.

## Introduction

Osteoarthritis (OA) affects more than 27 million Americans and is a leading cause of pain and disability [1]. There is currently no cure for OA [2]. The optimal treatment would be an OA disease-modifying therapy that can arrest the progressive degradation and eventual loss of articular cartilage in OA and improve symptomatic relief. Currently, most pharmacologic treatments are concentrated on secondary effects of the disease, such as relieving pain and improving joint function, but fail to address the evolving and complex nature of OA [3]. Commonly prescribed analgesics and nonsteroidal anti-inflammatory drugs (NSAIDs) provide symptomatic relief but do not have any demonstrated any beneficial effect on OA disease prevention or modification [4]. Furthermore, long-term use of these drugs has in some cases been associated with substantial gastrointestinal, renal and cardiovascular side effects [4]. Because the nature of OA likely requires decades-long treatment [5], novel therapies to combat this disease must be safe for clinical use over long periods of time.

Epigallocatechin 3-gallate (EGCG), a major bioactive polyphenol present in green tea, belongs to a group of food-derived products, termed *nutraceuticals*, with reported health benefits. Nutraceuticals have been suggested as safe alternatives or supplements to current pharmacologic therapies for OA [6,7]. EGCG exerts numerous health-promoting effects to counteract inflammation, aging and cancer [6]. In addition, EGCG has other reported effects particularly relevant to OA, such as inhibiting the production of inflammatory mediators such as nitric oxide, prostaglandin E_2_ (PGE_2_), cyclooxygenase-2 (COX-2), inducible nitric oxide synthase and interleukin (IL)-8 in human and equine chondrocytes *in vitro* [8,9]. *In vitro* studies also showed that EGCG inhibits mRNA and protein expression of matrix metalloproteinase (MMP)-1 and MMP-13 [10] and suppresses IL-1β-induced glycosaminoglycan release from cartilage by reducing the levels of A disintegrin and metalloproteinase with thrombospondin motifs 1 (ADAMTS1), ADAMTS4 and ADAMTS5 [11]. Furthermore, catechins from green tea inhibit the degradation of proteoglycan and type II collagen in bovine and human cartilage [12]. Also, green tea polyphenols added to drinking water reduce the incidence of collagen-induced arthritis and decrease the levels of COX-2 and tumor necrosis factor (TNF)-α in articular joints in mice [13]. However, the extent to which EGCG alters OA progression *in vivo* and improves OA-related symptoms, especially pain, has not been reported.

In this study, we addressed the question of whether EGCG could prevent progression of OA and relieve OA-associated pain in mice with posttraumatic OA induced by destabilization of the medial meniscus (DMM). To assess disease modification, we evaluated the integrity of the articular cartilage by using the following methods: (1) Safranin O staining and the Osteoarthritis Research Society International (OARSI) score; (2) immunohistochemistry of two crucial enzymes in OA progression, MMP-13 and ADAMTS5, as well as of cleaved aggrecan and type II collagen, as indicators of their activities; and (3) gene expression analysis of other proteolytic enzymes, including *Mmp1, Mmp2, Mmp3, Mmp8*; inflammatory cytokines (*Il1b*, *Tnfa*); and CBP/p300-interacting transactivator with ED-rich tail 2 (*Cited2*), a transcriptional regulator associated with the maintenance of cartilage integrity [14,15]. To assess symptom modification, we evaluated the effect of EGCG on pain relief by using pain behavioral assays and examining expression of pain markers and proinflammatory cytokines in the dorsal root ganglion (DRG).

## Methods

### Induction of osteoarthritis in mice and EGCG treatment

All studies were approved by the Albert Einstein College of Medicine Institutional Animal Care and Use Committee. DMM was established in adult C57BL/6 mice (males 5 to 6 months of age) by surgically transecting the medial meniscotibial ligament (MMTL) in the right hind limb [16]. Briefly, the joint capsule immediately medial to the patellar tendon was incised, followed by blunt dissection of the fat pad, to provide visualization of the MMTL of the medial meniscus. The MMTL was transected, leading to DMM. In the sham surgery, the MMTL was visualized but not transected. The joint capsule and skin were closed with sutures. Immediately after the DMM surgery, 100 μl of EGCG (25 mg/kg; Sigma-Aldrich, St Louis, MO, USA) dissolved in phosphate-buffered saline) or vehicle was administered via intraperitoneal injection once daily for 4 or 8 weeks. At 4 and 8 weeks postsurgery, groups of treated and control animals (*n* = 6/group) were selected for analysis as described below. The 25 mg/kg dose was chosen for intraperitoneal injection based on a dose–response experiment using 10 mg/kg to 50 mg/kg EGCG, in agreement with previous studies [17-19] (see Additional file 1: Figure S1 for details).

### Immunohistochemistry, Safranin O staining and OARSI score evaluation

At 4 and 8 weeks, groups of six animals were euthanized, and their hind limbs were fixed in formalin, decalcified in formic acid, embedded in paraffin and sectioned for histology and immunohistochemistry. Sections were incubated overnight at 4°C with antibodies against cleaved aggrecan (NITEGE; IBEX Technologies, Montreal, QC, Canada) and cleaved type II collagen (Col2-3/4 M; IBEX Technologies), MMP-13 (Abcam, Cambridge, UK) and ADAMTS5 (Abcam), followed by incubation with anti-mouse or anti-rabbit secondary antibody (Biocare Medical, Concord, CA, USA) and visualization with 3,3′-diaminobenzidine chromogen (Vector Laboratories, Burlingame, CA, USA). Negative controls were stained with irrelevant isotype-matched antibodies (Biocare Medical). Safranin O fast green staining was used to visualize proteoglycans in the articular cartilage. OA severity was evaluated with six sections of the articular cartilage for each mouse using the OARSI scoring system [20]. Immunostaining intensity for type II collagen or aggrecan cleavage epitopes was quantified by determining the reciprocal intensity of the stained articular cartilage matrix. Briefly, the light intensity value of six random locations within all three zones from the posterior to anterior direction of the femoral and tibial condyles of three sections per mouse was measured using the color picker in Adobe Photoshop (Adobe Systems, San Jose, CA, USA) [21]. Percentages of positive MMP-13 and ADAMTS5 chondrocytes were determined by counting the number of immunostained cells and dividing by the total number of chondrocytes visualized by using a hematoxylin counterstain (Vector Laboratories).

### Tactile sensitivity testing

Mice were acclimated for 30 minutes in individual chambers on top of a wire grid platform prior to von Frey testing. The plantar surface of the hind paw was stimulated with ascending force intensities of von Frey filaments (Stoelting, Wood Dale, IL, USA) to determine tactile sensitivity. A positive response was defined as a rapid withdrawal of the hind paw when the stimulus was applied, and the number of positive responses for each stimulus was recorded. Tactile threshold was defined as a withdrawal response to a given stimulus intensity in five of ten trials [22]. This threshold was calculated once per animal.

### Open-field behavioral test

Mice were acclimated to the test room for 30 minutes before open-field testing. They were placed in the center of individual Plexiglass square chambers (45 cm × 45 cm) and allowed to freely explore the chamber for the duration of the 6-minute test session. The movements of the mice were recorded with a video camera. Upon completion of the test, which was performed once per animal, each mouse was returned to its home cage [23]. Two observers blinded to treatment group assignments manually traced the mice’s movements to calculate the distance (in centimeters) the mouse traveled within the cage in 6 minutes and recorded the number of times each mouse reared (standing on its hind limbs) within the 6-minute period.

### Real-time PCR

The articular cartilage of sham or DMM mice treated with vehicle or EGCG for 4 weeks was harvested. For mice treated for 8 weeks, L3-L5 DRGs innervating the ipsilateral knee were collected and flash-frozen in liquid nitrogen. RNA was isolated using a QIAGEN RNeasy kit (QIAGEN, Valencia, CA, USA), and cDNA was synthesized using an iScript reverse transcriptase kit (Bio-Rad Laboratories, Hercules, CA, USA). Real-time PCR was performed in duplicate for each sample to determine relative gene expression, using glyceraldehyde 3-phosphate dehydrogenase as a housekeeping control, with the comparative cycle threshold method.

### Statistical analysis

The results are expressed as mean ± SD. Significance was determined using one-way analysis of variance and Sidak’s multiple-comparisons test, with GraphPad Prism software (GraphPad Software, La Jolla, CA, USA). *P* < 0.05 was considered as statistically significant.

## Results

### EGCG administration slows progression in early and midstage OA in DMM mice

**Figure 1:**
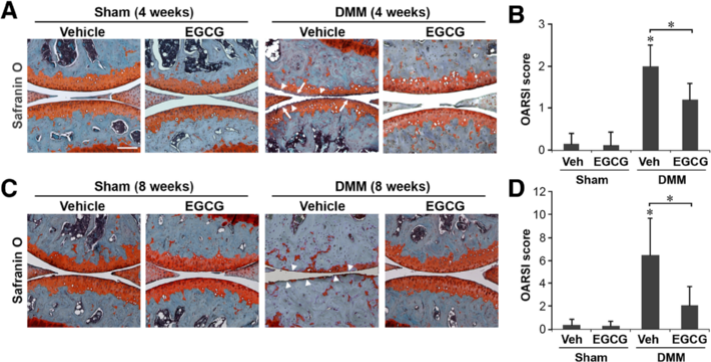
**Epigallocatechin 3-gallate administration slows progression in early and midstage osteoarthritis in mice that underwent surgical destabilization of the medial meniscus.** Mice underwent sham surgery or surgical destabilization of the medial meniscus (DMM), and cartilage specimens were treated with vehicle or epigallocatechin 3-gallate (EGCG). Safranin O stains and Osteoarthritis Research Society International (OARSI) scores of mice at 4 weeks **(A and B)** and 8 weeks **(C and D)** following surgery are shown (*P < 0.05 by analysis of variance; *n* = 6/group). Arrowheads indicate the areas of cartilage fibrillation or erosion, and arrows indicate loss of Safranin O staining.

To evaluate the efficacy of EGCG on DMM-induced OA initiation and progression, the structural integrity of the articular cartilage was examined by microscopy after Safranin O staining and OARSI evaluation. Four weeks after DMM, the articular cartilage in the DMM limb in the vehicle-treated mice exhibited mild OA pathological changes characterized by proteoglycan loss, cartilage fibrillation and an average OARSI score of 2.0 ± 0.5 (Figures 1A and 1B). In contrast, the cartilage in the DMM limb of EGCG-treated mice exhibited less proteoglycan loss and cartilage fibrillation, and the mean OARSI score (1.2 ± 0.4) was significantly lower compared to vehicle-treated controls (*P* < 0.05) (Figures 1A and 1B). Sham-operated mice that received either vehicle or EGCG treatment did not exhibit pathologic changes in the articular cartilage and had OARSI scores of 0.15 ± 0.25 and 0.12 ± 0.31, respectively (Figures 1A and 1B).

The vehicle-treated DMM mice exhibited more severe OA at 8 weeks than at 4 weeks, characterized by more pronounced proteoglycan loss and cartilage erosion, with an average OARSI score of 6.5 ± 3.2 (Figures 1C and 1D). In DMM mice treated with EGCG, OA severity was significantly lower (OARSI score, 2.1 ± 1.6; *P* < 0.05) compared to vehicle-treated DMM mice, pathological changes were limited to cartilage fibrillation and proteoglycan loss, and no cartilage erosion was observed. Sham-operated animals treated with vehicle control or EGCG exhibited no significant cartilage degradation, with average OARSI scores of 0.37 ± 0.5 and 0.29 ± 0.4, respectively (Figures 1C and 1D).

### EGCG administration reduced degradation of both type II collagen and aggrecan in articular cartilage matrix

**Figure 2:**
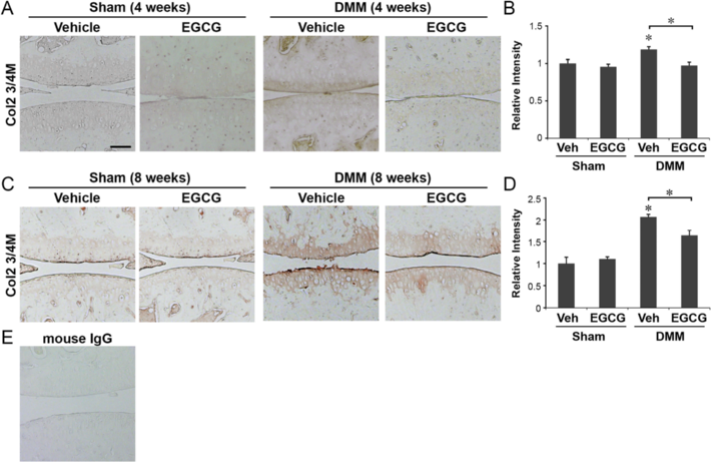
**Epigallocatechin 3-gallate administration reduced the degradation of type II collagen in the articular cartilage matrix.** Immunohistochemical staining of type II collagen cleavage epitope (Col2-3/4 M) and relative staining intensity of the articular cartilage matrix of mice that underwent sham operations or surgical destabilization of the medial meniscus (DMM) that were treated with vehicle or epigallocatechin 3-gallate (EGCG) at 4 weeks **(A and B)** and 8 weeks **(C and D)** following surgery (*P < 0.05 by analysis of variance; *n* = 6/group). Scale bar = 100 μM. **(E)** Representative staining of tissue sections with isotype control (mouse immunoglobulin G (IgG)).

Immunohistochemical staining showed that EGCG treatment strongly reduced the levels of the type II collagen cleavage epitope (Col2-3/4 M) in DMM mice compared to vehicle-treated DMM mice (Figure 2). On the basis of the immunostaining intensities of six randomly selected areas of the articular cartilage at 4 weeks following DMM, we found that type II collagen cleavage in vehicle-treated controls increased to 1.18-fold above sham-operated, vehicle-treated mice and was reduced to 0.97-fold in EGCG-treated animals (*P* < 0.05) (Figures 2A and 2B). At 8 weeks, the immunostaining intensities of the type II collagen cleavage epitope were 2.06-fold and 1.63-fold above sham-operated controls in vehicle and EGCG-treated DMM mice, respectively (*P* < 0.05) (Figures 2C and 2D). Sham-operated animals treated with vehicle or EGCG had no significant immunostaining for type II collagen degradation at 4 or 8 weeks (Figures 2A to 2D).

**Figure 3:**
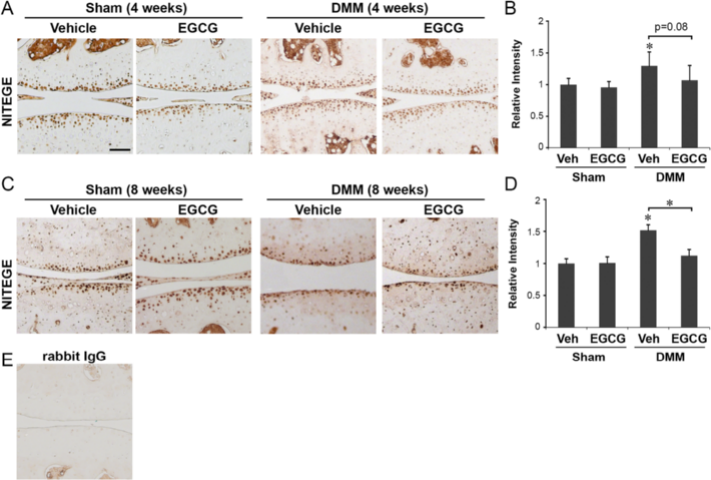
**Epigallocatechin 3-gallate administration reduced the degradation of aggrecan in the articular cartilage matrix.** Immunohistochemical staining of cleaved aggrecan (NITEGE) and relative staining intensity in the articular cartilage matrix of mice that underwent sham operations or surgical destabilization of the medial meniscus (DMM) that were treated with vehicle or epigallocatechin 3-gallate (EGCG) at 4 weeks **(A and B)** and 8 weeks **(C and D)** following surgery (*P < 0.05 by analysis of variance; *n* = 6/group). Scale bar = 100 μM. **(E)** Representative staining of tissue sections with isotype control (rabbit immunoglobulin G (IgG)).

Immunohistochemical staining similarly showed that EGCG treatment reduced the levels of cleaved aggrecan (NITEGE) in DMM mice compared to vehicle-treated DMM mice at 8 weeks (Figure 3). At 4 weeks after DMM, the intensity of aggrecan cleavage was not significantly different in the EGCG-treated mice compared to the vehicle control group (*P* = 0.08) (Figures 3A and 3B). At 8 weeks after DMM, the immunostaining intensity of cleaved aggrecan in EGCG-treated DMM mice was reduced to 1.12-fold compared to 1.51-fold in vehicle-treated mice (*P* < 0.05) (Figures 3C and 3D). Sham-operated animals treated with vehicle or EGCG did not exhibit significant immunostaining for cleaved aggrecan at 4 or 8 weeks (Figures 3A to 3D).

### EGCG administration reduced MMP-13 and ADAMTS5 levels in articular cartilage

Cartilage matrix degradation is mediated mainly by two major families of proteolytic enzymes: MMPs and ADAMTS [24]. In particular, MMP-13 is the most potent enzyme in cleaving type II collagen, the principal form in articular cartilage, whereas ADAMTS5 has been shown in mice to cleave aggrecan, the major cartilage proteoglycan [2]. Therefore, using immunohistochemistry, we examined whether reduction of MMP-13 and ADAMTS5 could underlie the chondroprotective effect of EGCG.

**Figure 4:**
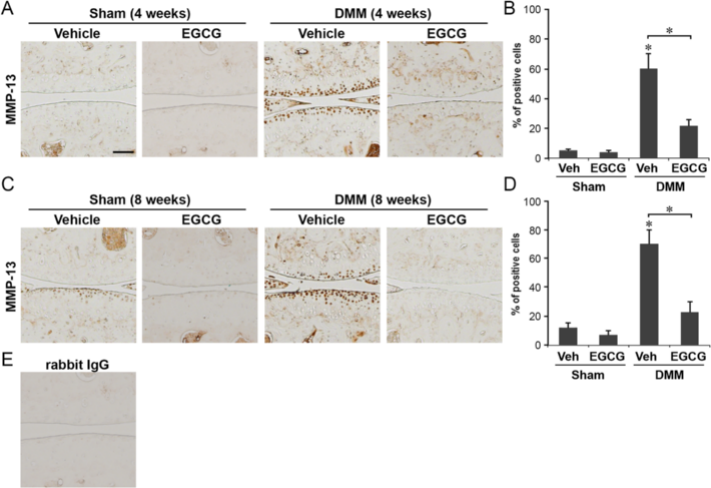
**Epigallocatechin 3-gallate administration reduced matrix metalloproteinase 13 levels in articular cartilage.** Immunohistochemical staining of matrix metalloproteinase 13 (MMP-13) and percentage of MMP-13-positive cells in the articular cartilage of mice that underwent sham operations or surgical destabilization of the medial meniscus (DMM) that were treated with vehicle or epigallocatechin 3-gallate (EGCG) at 4 weeks **(A and B)** and 8 weeks **(C and D)** following surgery (*P < 0.05 by analysis of variance; *n* = 6/group). Scale bar = 100 μM. **(E)** Representative staining of tissue sections with isotype control (rabbit immunoglobulin G (IgG)).

At 4 weeks following DMM, the percentage of MMP-13-positive cells was reduced from 60% in vehicle-treated mice to 22% in EGCG-treated mice (*P* < 0.05). MMP-13-positive chondrocytes in the vehicle-treated DMM mice were distributed in all three zones of the articular cartilage of the femoral and tibial condyles. In contrast, the MMP-13-positive chondrocytes in EGCG-treated mice were localized mainly in the middle and deep zones (Figures 4A and 4B). Levels of MMP-13-positive cells in the articular cartilage of EGCG-treated, sham-operated mice were at a level (4%) similar to that in the sham-operated, vehicle-treated mice (5%) (Figures 4A and 4B). At 8 weeks after DMM, the percentage of MMP-13-positive cells in the articular cartilage was reduced from 70% in vehicle-treated mice to 23% in EGCG-treated mice (*P* < 0.05). MMP-13-positive chondrocytes in the articular cartilage of EGCG-treated sham surgery animals were slightly lower (7%) than those in vehicle-treated controls (12%) (Figures 4C and 4D).

**Figure 5:**
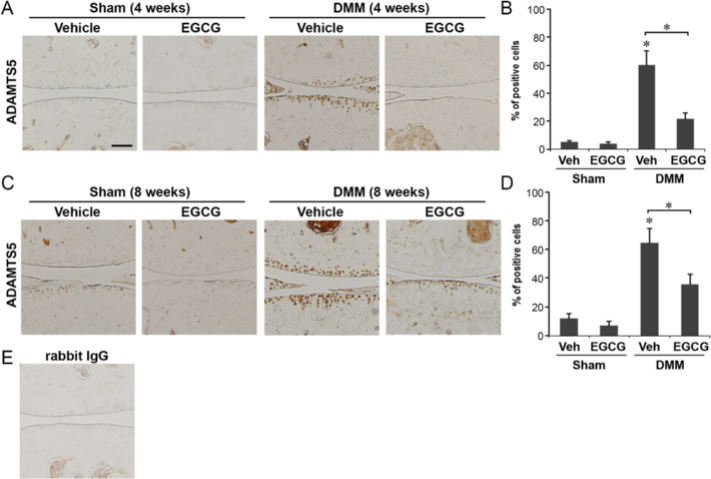
**Epigallocatechin 3-gallate administration reduced ADAMTS5 levels in the articular cartilage.** Immunohistochemical staining of A disintegrin and metalloproteinase with thrombospondin motifs 5 (ADAMTS5) and percentage of ADAMTS5-positive cells in the articular cartilage of mice that underwent sham operations or surgical destabilization of the medial meniscus (DMM) that were treated with vehicle or epigallocatechin 3-gallate (EGCG) at 4 weeks **(A and B)** and 8 weeks **(C and D)** following surgery (*P < 0.05 by analysis of variance; *n* = 6/group). Scale bar = 100 μM. **(E)** Representative staining of tissue sections with isotype control (rabbit immunoglobulin G (IgG)).

Similarly, at 4 weeks following DMM surgery, EGCG reduced the percentage of ADAMTS5-positive cells from 61% in vehicle-treated mice to 22% in EGCG-treated mice (*P* < 0.05) (Figures 5A and 5B). EGCG treatment appeared to exert no measurable effect on the levels of ADAMTS5 in chondrocytes in the articular cartilage in the sham-operated animals treated either with vehicle (5%) or with EGCG (4%) (Figures 5A and 5B). In the articular cartilage at 8 weeks following DMM, the percentages of ADAMTS5-positive cells were 63% in vehicle-treated mice and 37% in EGCG-treated mice (*P* < 0.05) (Figures 5C and 5D). EGCG did not significantly alter the percentage of ADAMTS5-positive cells in sham-operated mice (Figures 5C and 5D). These data suggest that EGCG treatment improves the integrity of the articular cartilage by preserving both collagen and aggrecan components in posttraumatic OA mice and that the chondroprotective effects exerted by EGCG are mediated, at least in part, by reducing MMP-13 and ADAMTS5 levels.

### EGCG treatment results in a chondroprotective gene expression profile

**Figure 6:**
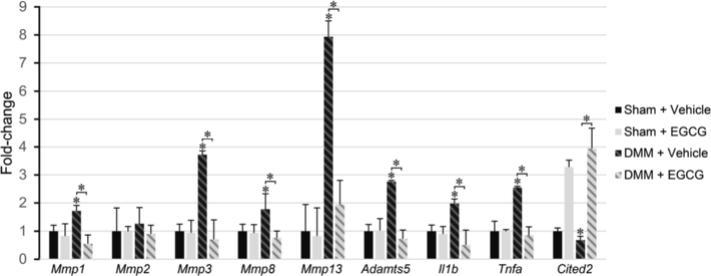
**Epigallocatechin 3-gallate treatment results in a chondroprotective gene expression profile.** Relative gene expression levels of catabolic enzymes, inflammatory cytokines and the matrix metalloproteinase (MMP) regulator CBP/p300-interacting transactivator with ED-rich tail 2 (*Cited2*) in the articular cartilage of mice that underwent sham operations or surgical destabilization of the medial meniscus (DMM) that were treated with vehicle or epigallocatechin 3-gallate (EGCG) at 4 weeks following surgery (*P < 0.05 by analysis of variance; *n* = 5/group). *Adamts5*, A disintegrin and metalloproteinase with thrombospondin motifs 5; *Il1b*, interleukin 1 beta; *Tnfa*, Tumor necrosis factor alpha.

To further understand the mechanism underlying the effects of EGCG on cartilage integrity, we examined the expression of genes encoding proteins with functions closely related to cartilage homeostasis, in addition to MMP-13 and ADAMTS5, including proteolytic enzymes MMP-1, MMP-2, MMP-3 and MMP-8; the MMP-repressing transcriptional regulator CITED2; and proinflammatory cytokines, IL-1β and TNF-α. EGCG treatment significantly reduced the mRNA levels of *Mmp1, Mmp3, Mmp8, Mmp13*, *Adamts5*, *Il1b* and *Tnfa* and increased *Cited2* mRNA in the articular cartilage of DMM mice compared to vehicle-treated mice (*P* < 0.05 in all cases) (Figure 6) in the articular cartilage. The data demonstrate that EGCG exerts a broad spectrum of anti-inflammatory and anticatabolic effects, involving cytokines, inflammatory mediators, proteolytic enzymes and transcriptional regulators.

### EGCG reduces osteoarthritis-related pain symptoms

**Figure 7:**
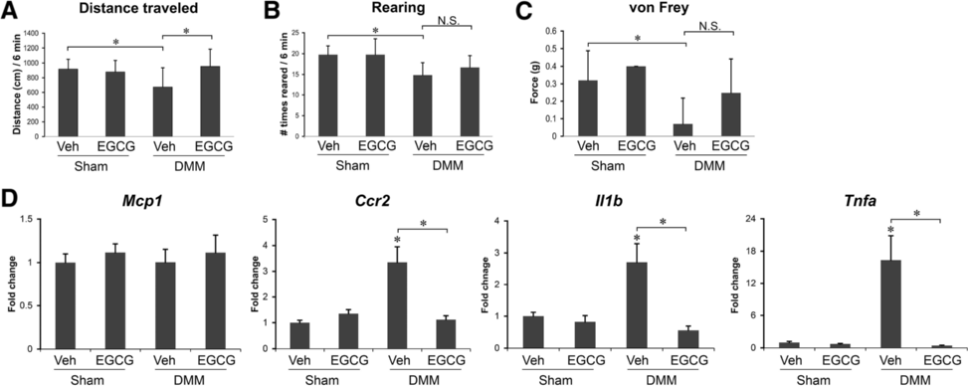
**Epigallocatechin 3-gallate reduces osteoarthritis-related pain symptoms.** Behavior assessments of mice that underwent sham operations or surgical destabilization of the medial meniscus (DMM) that were treated with vehicle or epigallocatechin 3-gallate (EGCG) at 8 weeks after DMM surgery. **(A)** Distance traveled. **(B)** Number of times mice reared in an open cage within 6 minutes. **(C)** von Frey testing (mechanical allodynia). **(D)** Gene expression of monocyte chemoattractant protein 1 (*Mcp1*), chemokine receptor 2 (*Ccr2*), interleukin 1 beta (*Il1b*) and tumor necrosis factor alpha (*Tnfa*) in the ipsilateral L3-L5 dorsal root ganglion (*P < 0.05 by analysis of variance; *n* = 6/group). N.S., Not significant.

The progression of OA is accompanied by secondary clinical symptoms, most prominently pain [25,26]. At 8 weeks after DMM, vehicle-treated DMM mice exhibited reductions in distance traveled (*P* < 0.05) (Figure 7A), rearing (standing on hind limbs) (*P* < 0.05) (Figure 7B) and response threshold to mechanical stimuli (tactile hypersensitivity) (*P* < 0.05) (Figure 7C), compared to sham-operated controls. EGCG treatment suppressed one of these pain responses: distance traveled was the same as in sham-operated mice (*P* < 0.05) (Figure 7A). There was no significant difference observed in rearing (Figure 7B) or tactile sensitivity of the paw (Figure 7C) in EGCG-treated mice.

The chemokine monocyte chemoattractant protein (MCP)-1 and its receptor, chemokine receptor 2 (CCR2) in the DRG, are central to the development of pain associated with OA. Increased mRNA levels of *Mcp1* and *Ccr2* in the ipsilateral DRG at 8 weeks after DMM are causally related to pain-related behaviors [26]. In our study, in vehicle-treated DMM mice at 8 weeks following surgery, *Mcp1* gene expression in the ipsilateral DRG remained unchanged (Figure 7D), whereas the mRNA levels of its receptor, *Ccr2*, and two chronic pain-associated proinflammatory cytokines, *Il1b* and *Tnfa*, were significantly upregulated (*P* < 0.05 in all cases) (Figure 7D). In the EGCG-treated animals, the levels of *Ccr2*, *Il1b* and *Tnfa* mRNA were similar to those observed in sham-operated mice and significantly reduced compared to those in vehicle-treated controls (*P* < 0.05) (Figure 7D).

## Discussion

There is currently no therapeutic agent with a clearly demonstrated ability to modify the course of OA [27]. In this study, we provide direct *in vivo* evidence that administration of EGCG slows the progression of posttraumatic OA in the DMM mouse model. EGCG-treated mice exhibited less cartilage erosion and proteoglycan loss, improved preservation of type II collagen and aggrecan and reduced levels of MMP-13 and ADAMTS5, two crucial proteolytic enzymes involved in the degradation of those matrix components [24]. Although the efficacy of EGCG in human OA has not yet been tested in controlled trials, our findings provide fundamental evidence and a sound rationale for advancing EGCG-based treatments toward clinical application.

The chondroprotective effects of EGCG on attenuating inflammation and catabolic activity have been established *in vitro* in studies using human chondrocytes [8-10,28-32], synovial fibroblasts [33-36] and human and bovine cartilage explants [12], as well as in rheumatoid arthritis animal models [37-41]. Consistent with these studies, our present study demonstrates that EGCG exerts broad chondroprotective effects in a posttraumatic OA mouse model *in vivo* by suppressing the expression of genes encoding inflammatory cytokines IL-1β and TNF-α and multiple cartilage-degrading enzymes, including MMPs 1, 3, 8 and 13 and ADAMTS5, as well as by inducing gene expression of the MMP-repressing transcriptional regulator *Cited2*. In our previous study, we demonstrated that, in response to moderate mechanical loading, *CITED2* represses *MMP-1* and *MMP-13* gene transcription *in vitro* [14] and *in vivo* [15]. Of note, EGCG elevated *Cited2* expression in OA (DMM) as well as non-OA (sham) articular cartilage, suggesting that it may play cartilage-protective roles under both physiological and OA pathological conditions. The *in vivo* evidence provided in this study, together with a well-established chondroprotective effect demonstrated in previous studies, supports the concept that EGCG may be an effective chondroprotective agent for OA treatment.

In this study, we also provide evidence for an OA-related pain-relieving effect of EGCG in a posttraumatic OA mouse model. OA pain can be triggered by joint movement and typically results in diminished use and reduced joint mobility [25]. Patients with OA also have lower mechanical stimuli pain-sensing thresholds, suggesting that central sensitization also contributes to OA-related pain [42]. In our study, EGCG-treated DMM mice exhibited increased locomotor behavior (that is, distance traveled) compared to vehicle-treated mice, suggesting an improvement in OA-related pain. Of note, there was no significant difference in rearing or tactile sensitivity of the paw in EGCG-treated mice compared to vehicle controls. Because rearing (standing on the hind limbs) and tactile sensitivity are both measurements related to mechanical sensitivity of the limbs due to OA [26], this suggests that the improvement of pain by EGCG may not rely simply on the mechanical sensitivity of the diseased limb; however, it merits further study.

Interactions between neuropathic pathways and OA tissues influence the development of pain behaviors and alterations in gene transcription and protein expression in the sensory neurons of the DRG [43-46]. A previous study suggested that MCP-1 and CCR2 mediate pain development in OA mice, and the investigators reported a transient upregulation of MCP-1 and CCR2 at 8 weeks following DMM surgery [26]. To understand the mechanisms underlying the analgesic effects of EGCG, we examined expression of genes encoding the chemokine MCP-1 and its receptor CCR2, as well as the chronic pain-related proinflammatory cytokines IL-1β and TNF-α, in the DRG. In our DMM model, we did not find *Mcp1* upregulation in the DRG compared to sham-operated animals at 8 weeks, but elevated *Ccr2* mRNA, which together with increased levels of *Il1b* and *Tnfa* mRNA, suggests a receptor-oriented MCP-1/CCR2 pain-sensitizing mechanism. Interestingly, in EGCG-treated DMM mice, gene expression of *Ccr2* is significantly lower than in vehicle controls. This fact, together with reduced mRNA levels of *Il1b* and *Tnfa*, provides supporting evidence that EGCG exerts effects on pain-related disease modification by targeting the pain-associated mediators and cytokines in the pain sensitization pathway, in addition to structure-modifying effects through reducing OA severity. Consistent with our findings, a previous study demonstrated that intravenous infusion of EGCG improved pain symptoms in chronically injured spinal cord of adult rats [47].

EGCG has shown promise in clinical trials for the treatment of various cancers and cardiovascular and neurodegenerative disorders [48-52]. Furthermore, the chondroprotection of EGCG has been well established *in vitro* and *in vivo*, including in rheumatoid arthritis animal models [8-10,28-41,53], providing a solid foundation for further exploration of its therapeutic potential in preclinical and clinical studies. One advantage of using nutraceutical-based treatments such as EGCG is that they exhibit favorable safety profiles. Early studies demonstrated that EGCG was nontoxic to human chondrocytes [8]. In humans, daily doses of 800 mg of EGCG for 4 weeks are safe and well tolerated [54]. EGCG is mostly absorbed by the small intestine and may undergo gastrointestinal inactivation [54]. Therefore, oral administration of EGCG may reduce its bioavailability. Accordingly, in our study, we chose to administer EGCG systemically via intraperitoneal injection, which leads to higher bioavailability compared to oral consumption [54]. In future trials, researchers should consider optimizing EGCG bioavailability when given orally.

Destabilization of the medial meniscus is a commonly used surgically induced OA mouse model. In this model, OA results primarily from altered joint biomechanics and pathologic changes, including cartilage destruction, subchondral bone thickening and osteophyte formation, similar to those observed in human OA [16]. In the present study, we focused on the effects of EGCG on moderate to severe OA and therefore chose the 8-week experimental period because, based on previous studies [16] and also confirmed in this study, moderate to severe OA develops reproducibly at this time point following DMM surgery in mice. A study with a longer injury and treatment period is needed to evaluate the efficacy of EGCG on OA progression in severe and late-stage posttraumatic OA in mice. One limitation of this acute mouse trauma model is that it may not represent the more slowly progressive human OA such as that seen during aging. Further studies undertaken to investigate the efficacy of EGCG on other relevant OA models, such as spontaneous or aging-related OA, are of interest. In this study, we show evidence for the beneficial effects of EGCG on disease modification and symptom modification in mice with posttraumatic arthritis. Further studies addressing whether EGCG would have a clinical impact on OA are clearly needed to advance EGCG-based treatments toward applicability in humans.

Accumulating evidence indicates that EGCG exerts a variety of biological effects such as anti-inflammatory, antioxidative and anticatabolic effects on chondrocytes [8-10,28-32,55], suggesting that the mechanism which couples these different responses to EGCG may involve a common receptor. Tachibana *et al*. identified a 67 kDa nonintegrin cell surface laminin receptor that confers EGCG responsiveness to cancer cells at physiologically relevant concentrations [56]. Researchers in a subsequent study identified the motif to which EGCG binds to this receptor [57]. It will be interesting to uncover whether such a receptor or similar mechanism may exist in chondrocytes and, if it does, whether it plays a required role in mediating the effects of EGCG on chondrocytes.

Our study demonstrates significant efficacy of EGCG in disease and symptom modification of posttraumatic OA, and combining EGCG with other potential therapeutic agents may further enhance its efficacy. Recent studies show that, in cultured equine chondrocytes *in vitro*, EGCG and avocado/soybean unsaponifiables (ASU) individually inhibited IL-1β- and TNF-α-induced expression of COX-2 and PGE_2_; the combination of EGCG and ASU achieved synergistic effects on the suppression of COX-2 expression and PGE_2_ production [9]. Furthermore, a combination therapy of methotrexate and EGCG, compared to each treatment used individually, exerted a more profound reduction of the gene expression of proinflammatory cartilage cytokines (*TNF-α* and *IL-6*) and potentiated the antiarthritic (decrease in hind paw volume) and antioxidant effects [39]. Together, these studies suggest that the chondroprotective effect of EGCG may be amplified when used with other antiarthritic agents in the management of OA.

## Conclusions

Taken together, the results of this study provide the first evidence in an OA animal model that EGCG provides significant efficacy in arresting OA disease progression and exerts a substantial effect in OA pain relief. Our study suggests that the effect of EGCG on OA disease modification may be due to the action of modulating a broad spectrum of molecules that are critical for cartilage homeostasis. The effect of EGCG on symptom modulation in relieving OA-related pain involves suppression of the pain marker CCR2 and chronic pain-related proinflammatory cytokines in the DRG, in addition to reduced disease progression.

## Additional file

Additional file 1: Figure S1. Dose-dependent effects of EGCG on *Mmp13* and *Adamts5* in DMM mice. EGCG was administered intraperitoneally at various doses (0, 10, 25, 50 mg/kg) daily for 3 days starting immediately after destabilization of the medial meniscus (DMM) surgery in mice (C57BL/6, 6-month-old males, *n* = 4/group). Expression of genes encoding the proteolytic enzymes MMP-13 and ADAMTS5 was assessed by real-time PCR of total RNA isolated from the articular cartilage. EGCG at 25 mg/kg and 50 mg/kg decreased the levels of both *Mmp13* and *Adamts5* mRNA by 50% to 60% relative to vehicle control (**P* < 0.05, one-way ANOVA with Tukey’s *post hoc* test), whereas animals treated with 10 mg/kg showed a significant reduction of *Mmp13* mRNA, but not *Adamts5* mRNA. Because there was no statistical difference between the 25 mg/kg and 50 mg/kg treatment groups, the lower dose (25 mg/kg) was chosen for the present study.

## Abbreviations

ADAMTS: A disintegrin and metalloproteinase with thrombospondin motifs
CCR: Chemokine receptor
CITED2: Cbp/p300 interacting transactivator with ED-rich tail 2
COX-2: Cyclooxygenase-2
DMM: Destabilization of the medial meniscus
DRG: Dorsal root ganglion
EGCG: Epigallocatechin 3-gallate
IL-1β: Interleukin-1β
MCP: Monocyte chemoattractant protein
MMP: Matrix metalloproteinase
NSAID: Nonsteroidal anti-inflammatory drug
OA: Osteoarthritis
OARSI: Osteoarthritis Research Society International
PGE_2_: Prostaglandin E_2_
TNF-α: Tumor necrosis factor-α
