# Correction to: Green tea polyphenol treatment is chondroprotective, anti-inflammatory and palliative in a mouse posttraumatic osteoarthritis model

**DOI:** 10.1186/s13075-018-1791-9

**Published:** 2019-01-03

**Authors:** Daniel J. Leong, Marwa Choudhury, Regina Hanstein, David M. Hirsh, Sun Jin Kim, Robert J. Majeska, Mitchell B. Schaffler, John A. Hardin, David C. Spray, Mary B. Goldring, Neil J. Cobelli, Hui B. Sun

**Affiliations:** 1 Department of Orthopaedic Surgery, Albert Einstein College of Medicine, 1300 Morris Park Ave, Bronx, NY 10461 USA; 2 Department of Radiation Oncology, Albert Einstein College of Medicine, 1300 Morris Park Ave, Bronx, NY 10461 USA; 3 Department of Neuroscience, Albert Einstein College of Medicine, 1410 Pelham Pkwy S, Bronx, NY 10461 USA; 4 Department of Biomedical Engineering, The City College of New York, 160 Convent Ave, New York, NY 10031 USA; 5 Tissue Engineering, Regeneration and Repair Program, Hospital for Special Surgery, 535 East 70th Street, New York, NY 10021 USA

**Correction to: Arthritis Res Ther**

**https://doi.org/10.1186/s13075-014-0508-y**

Following publication of the original article [1], the authors reported an error in Figs. 2C and 5C. The incorrect and the correct figures are displayed below.




