# Supplementary material for: Green tea polyphenol treatment is chondroprotective, anti-inflammatory and palliative in a mouse posttraumatic osteoarthritis model
Source: Arthritis Res Ther. 2014 Dec 17;16(6):508. doi: 10.1186/s13075-014-0508-y (PMC4342891; doi:10.1186/s13075-014-0508-y)
Supplement: Additional file 1: Figure S1. — Dose-dependent effects of EGCG on Mmp13 and Adamts5 in DMM mice. EGCG was administered intraperitoneally at various doses (0, 10, 25, 50 mg/kg) daily for 3 days starting immediately after destabilization of the medial meniscus (DMM) surgery in mice (C57BL/6, 6-month-old males, n = 4/group). Expression of genes encoding the proteolytic enzymes MMP-13 and ADAMTS5 was assessed by real-time PCR of total RNA isolated from the articular cartilage. EGCG at 25 mg/kg and 50 mg/kg decreased the levels of both Mmp13 and Adamts5 mRNA by 50% to 60% relative to vehicle control (*P < 0.05, one-way ANOVA with Tukey’s post hoc test), whereas animals treated with 10 mg/kg showed a significant reduction of Mmp13 mRNA, but not Adamts5 mRNA. Because there was no statistical difference between the 25 mg/kg and 50 mg/kg treatment groups, the lower dose (25 mg/kg) was chosen for the present study. [file 13075_2014_508_MOESM1_ESM.doc]

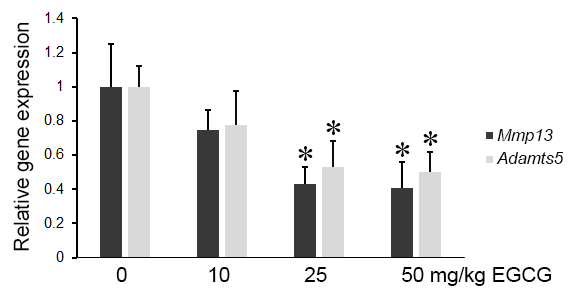


**Additional file 1: Figure S1: Dose-dependent effects of EGCG on *Mmp13* and *Adamts5* in DMM mice.** EGCG was administered intraperitoneally at various doses (0, 10, 25, 50 mg/kg) daily for three days starting immediately after destabilization of the medial meniscus (DMM) surgery in mice (C57BL/6, 6-month-old male, n=4/group). Expression of genes encoding the proteolytic enzymes MMP-13 and ADAMTS5 was assessed by real-time PCR of total RNA isolated from the articular cartilage. EGCG at 25 mg/kg and 50 mg/kg decreased the levels of both *Mmp13* and *Adamts5* mRNA by 50 – 60% relative to vehicle control (*=p<0.05, one-way ANOVA, Tukey posthoc test), while animals treated with 10 mg/kg showed a significant reduction of *Mmp13* mRNA, but not *Adamts5* mRNA. Because there was no statistical difference between the 25 mg/kg and 50 mg/kg treatment groups, the lower dose (25 mg/kg) was chosen for the current study.
